# No lack of regulatory B cells in patients with Multiple Sclerosis

**DOI:** 10.1186/1479-5876-10-S3-O4

**Published:** 2012-11-28

**Authors:** Laure Michel, Mélanie Chesneau, Philippe Manceau, Alexandra Garcia, Marion Salou, Annie Elong Ngono, Annaïck Pallier, Marylène Jacq-Foucher, Fabienne Lefrère, Sandrine Wiertlewski, Jean-Paul Soulillou, Nicolas Degauque, David-Axel Laplaud, Sophie Brouard

**Affiliations:** 1 INSERM, UMR 1064, Nantes, France; 2 CHU Nord Laennec, Service de Neurologie, Nantes, France; 3 CHU de Nantes, ITUN, Nantes, France

## Background

Recent data support a prominent role for B cells in MS physiopathology. Recently it has emerged that subsets of B cells secreting IL-10 negatively regulate disease symptoms in Experimental Autoimmune Encephalomyelitis (EAE). However, the involvement of such regulatory B cells in MS remains unclear.

## Aim

We aimed to study the frequency, phenotype and function of regulatory B cells in MS patients as compared to Healthy Volunteers (HV).

## Methods

Sixty-three untreated MS patients and 58 HV were included in this study. IL-10 secretion by B cells and phenotype of IL-10^+^ B cells were studied after 5h (B10 cells) and 48h of stimulation (B10pro cells) by CD40L and ODN. Coculture assays with prestimulated B cells and responding CD4^+^CD25^-^ T cells were performed for 3 days.

## Results

No significant difference was found either for IL-10 secretion ability of B cells after 5h or 48h of stimulation. The analysis of B10pro cells phenotype revealed mainly a memory phenotype in MS and HV, even if both naïve and immature subsets were also able to secrete IL-10. Prestimulated B cells from MS inhibited CD4^+^CD25^-^ T cell proliferation in the same manner than HV by a contact dependent mechanism, independently of IL-10 and TGF-β secretion.

## Conclusion

Altogether, our data show that regulatory B cells have a conserved frequency, phenotype and function in the blood of patients with MS suggesting that B cells do not contribute to the physiopathology of the disease.

## Acknowledgements

This work was funded by ARSEP.

